# The Impact of Patient and Professional Users’ Involvement in Implementation for Virtual Reality in Hospitalised Palliative Cancer Patients in a German Cancer Centre—A Qualitative Analysis

**DOI:** 10.3390/healthcare14131876

**Published:** 2026-06-26

**Authors:** Christina Gerlach, Laura Haas, Melanie Guenther, Kate Binnie, Jonah Lantelme, Julia Thiesbonenkamp-Maag, Bernd Alt-Epping, Cornelia Wrzus

**Affiliations:** 1 Department of Palliative Medicine, Heidelberg University Hospital, Im Neuenheimer Feld 305, 69120 Heidelberg, Germany; laura.haas01@gmail.com (L.H.); melanie.guenther95@gmx.de (M.G.); jonah.lantelme@stud.uni-heidelberg.de (J.L.); julia.thiesbonenkamp-maag@med.uni-heidelberg.de (J.T.-M.); bernd.alt-epping@med.uni-heidelberg.de (B.A.-E.); 2 Wolfson Palliative Care Research Centre, Hull York Medical School, Hull HU6 7RX, UK; hyib9@hyms.ac.uk; 3 Psychological Institute and Network Aging Research, Ruprecht Karls University of Heidelberg, Bergheimer Str. 20, 69115 Heidelberg, Germany; wrzus@psychologie.uni-heidelberg.de

**Keywords:** digital health, virtual reality, palliative care, oncology, individualised therapy, patient and public involvement

## Abstract

**Highlights:**

**What are the main findings?**

- The study underlines the importance of patients’, relatives’, and healthcare professionals’ perspectives in the implementation strategy for individualised VR.
- Staff attitudes can pose challenges, though various strategies were discussed to overcome rejectionist perspectives.
- High organisational demands and staff deployment were identified as major weaknesses in implementation.
- Patients and relatives expressed concerns about data protection when using private VR content.
- Despite limited evidence in previous studies, concerns about the potential emotional burden on patients persisted.

**What are the implications of the main findings?**

- Concerns among patients, relatives, and clinical staff should be addressed through active listening and clear information, as unaddressed worries may otherwise obscure evidence and hinder implementation
- Individualised VR cannot be implemented in palliative care without additional staff resources and consideration of data security.

**Abstract:**

**Background:** Virtual reality (VR) is a promising technology for the relief of physical and psychosocial burdens. We found that individualised VR videos were well tolerated and accepted and seemed to have a stronger effect on well-being and emotional connection than standardised VR in cancer inpatients under palliative care. For implementation, it is important to actively involve patients, as their input helps to ensure that the VR intervention meets their needs, thus making it more likely to be accepted and effective in practice, while balancing the needs of healthcare professionals. **Aim:** Exploration of patients’ and healthcare professionals’ perspectives on best practice VR intervention implementation. **Design:** Workshop-based 360° focus group using a strengths–weaknesses–opportunities–threats (SWOT) model and deductive/inductive qualitative analysis with a ‘framework’ approach. **Setting/participants:** The focus group took place at the National Centre for Tumour Therapy of a German university hospital. Participants were a local doctor (1) and nurses (3) with VR experience, the cooperating patient advisory board of the study (2), and members of a regional self-help group (3). **Results:** Eighteen subthemes were identified in the SWOT model. While there was agreement on the ‘strength of distraction’ and ‘opportunities of individualised VR’, concerns remained regarding data protection when using private VR content. There was an argument about gatekeeping by relatives worried about mental distress in patients immersing in home or family VR scenes. In contrast, many ideas were discussed regarding how to overcome rejectionist staff attitudes. However, the high organisational time and staff deployment were addressed as major weaknesses. **Conclusions:** Involving patient stakeholders and healthcare professionals in the planning of the implementation strategy revealed several issues that require attention. In particular, information needs to be provided not only to patients but also to relatives and hospital staff, alongside ensuring data protection and adequate staffing. **Trial registration:** Registered at German Clinical Trials Register (Deutsches Register Klinischer Studien; DRKS); registration number: DRKS00032172; registration date: 11 July 2023.

## 1. Introduction

As their disease progresses, palliative care cancer patients often experience a significant burden of symptoms, which impairs their well-being and quality of life [1]. They must adjust to irreversibly changed life circumstances and are also confronted with the end of their lives. In addition to physical symptoms of illness, many experience psychological and existential distress as a result. For example, patients with advanced cancer report a significantly higher level of psychological distress [2]. Significant psychosocial consequences are also reflected in the form of psychiatric and psychosocial morbidity as well as a high prevalence of psychological symptoms [3]. Accordingly, patients receiving palliative care have an increased risk of severe psychiatric complications, such as anxiety and depression [4,5]. Therefore, it seems crucial to address these psychological challenges and improve psychological well-being. To address this, psychological interventions serve as an important complement to medical care, providing holistic support for palliative care patients at the end of their lives.

Patients requiring inpatient palliative care are exposed not only to the symptoms of their illness, but also to the stress and isolation associated with hospitalisation [6]. For many patients, the wish to be and die at home is central [7]. However, to receive appropriate medical care and to address the complexity of medical problems that may arise in palliative situations, hospital care is often required [8]. Virtual reality (VR) may bridge the gap between the patient’s wish to be at home and the need for hospitalisation.

VR enables patients to immerse themselves in simulated environments that resemble real-world objects and events, creating a sense of “presence” within the virtual world [9]. By shielding users from real stimuli and replacing them with simulated sensory input, VR can provide distraction from physical and psychological burdens. In cancer care, VR has been shown to benefit patients in stressful or painful situations [10], although evidence remains limited for palliative cancer patients [11,12]. VR interventions are typically delivered via head-mounted displays (HMDs) and often use standardised nature scenes to distract patients during medical procedures or for symptom management [13]. Systematic reviews indicate that VR is generally feasible, safe, and acceptable in palliative settings [11,12,13,14,15,16]. VR interventions have been shown to improve well-being and quality of life and reduce symptom burden [10,11,16,17]. Beyond symptom relief, VR may address psychological and spiritual dimensions of palliative care, for example by enabling patients to experience familiar or personally meaningful environments and thereby supporting a sense of normality and “being at home” [18,19]. While most approaches rely on pre-selected content, individualised VR, allowing patients to choose from available options, has shown promise [20,21,22]. Preliminary evidence further suggests that such tailored, meaningful VR experiences may offer additional benefits for both physical and psychological symptoms [16,23]. In this project, an even more individualised approach was applied, with VR content specifically produced and tailored to each patient’s preferences and needs.

Throughout the project, we collaborated with the patient advisory board of the National Centre for Tumour Diseases (NCT), from protocol development through recruitment, interviews and conduct of the trial, to data analysis and publication [24]. First, patients’ and relatives’ expected benefits and concerns regarding VR and individualised content, such as representations of their own home, were explored in comprehensive interviews. Based on the findings of this study [25], a clinical feasibility trial using individualised VR was adapted to better meet the needs of palliative care patients [26].

From the outset, the project was designed to be completed with a patient and public involvement (PPI) initiative to support the co-development of implementation strategies for individualised VR interventions in palliative cancer care. Recognizing that highly standardised research conditions may not adequately reflect the diverse needs, preferences, and clinical circumstances of patients receiving—or professionals delivering—palliative care, the study sought to integrate the perspectives of both healthcare professionals and lay users. To achieve this, a workshop-based focus group was conducted to explore views on the implementation of individualised VR interventions and to identify practical recommendations for their future use in clinical practice. To capture a broad range of perspectives, the workshop brought together researchers, healthcare professionals, patients, and representatives of patient organisations. The findings of this process are reported below.

## 2. Methods

After completion of the interviews and the intervention trial [24,25,26], the final PPI focus group, which is described here, was conducted to discuss the findings and implications for implementation.

### 2.1. Methodological Orientation and Theory

This research is informed by critical realism, which assumes that reality exists independently of human perception while recognizing that our understanding of that reality is mediated through individuals’ experiences, interpretations, and social contexts [27,28]. The study adopted a pragmatic qualitative approach aimed at generating practically useful insights to inform future intervention development and implementation. A focus group was considered more appropriate than individual interviews to support the patient perspective by direct exchange of views and ideas with healthcare professionals in a safe environment. The focus group was prepared, conducted, and analysed by a working group of the department of palliative medicine at a German university hospital with a worldwide reputation in oncology. The members were the authors C.G. (MD and MSc in Palliative Care; in the following referred to as MOD1), M.G. (PhD candidate in Neurosciences; MOD2), J.L. (MD candidate; MOD3), J.T.-M. (PhD in Anthropology; MOD4), L.H. (MSc in Developmental and Clinical Psychology), and C.W. (Professor for Psychological Aging Research). The program schedule was planned and prepared based on the literature [29,30] and consultation with colleagues experienced in PPI. No pilot testing was performed.

A content analysis [31] was applied supported by the ‘framework approach’ to analyse the data from the focus group [32,33]. A ‘framework’ is an analytical tool to analyse descriptive accounts that summarise phenomena across individuals. It utilises five related steps: familiarisation, indexing and sorting, developing categories, data summary and display, and construction and identification of linkages between themes. To structure the workshop and guide the initial analysis, a ‘strengths, weaknesses, opportunities, threats’ (SWOT) approach was used. As a widely used framework for reflecting on strengths, weaknesses, opportunities, and threats in development processes, it was considered suitable for supporting patients’ leadership role in further developing the VR intervention while also balancing the needs of healthcare professionals. All methods presented herein are reported according to the COREQ guideline for qualitative research [34].

### 2.2. Sample and Setting

Participants of the focus group were purposively sampled according to their role: nurses and doctors caring for patients participating in the virtual reality study or otherwise experienced with VR in healthcare, members of the patient advisory board who contributed their advice to the protocol development and study conduct [24,25,26], and members of patient aid associations. They were informed and asked regarding their willingness and availability to participate in the focus group. Based on their feedback, the focus group was scheduled, and two reminders were sent during the preceding week.

### 2.3. Data Collection

The focus group took place in a seminar room of the NCT in Heidelberg, Germany, 10 June 2024.

After a welcome and a short introduction of all moderators and participants, all participants provided written informed consent and agreed to the voice recording of the focus group. All information on the VR study as well as questions to the audience were presented to the focus group using Microsoft PowerPoint slides. Prior to the beginning of the SWOT rounds, MOD1 and MOD2 provided detailed information about (1) the rationale and aims of the VR study, and (2) the results of the prior working packages on patients’ and relatives’ wishes and concerns regarding VR in hospital and their actual experiences [25,26]. Afterwards, participants were invited to share any remaining questions, as well as additional ideas and suggestions regarding the implementation of the VR intervention. Field notes and transcripts of the focus group remained with the moderators and were subsequently used for data analysis. The schedule is detailed in Table 1.

### 2.4. Data Analysis

**Figure 1:**
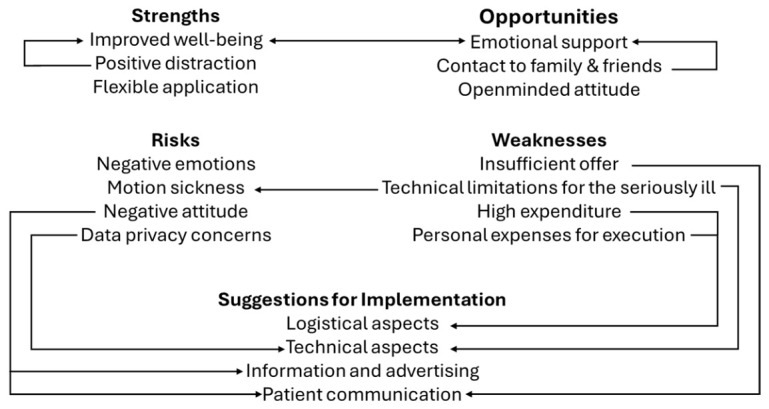
The framework analysis generated 5 overarching categories based on the SWOT framework (strengths, weaknesses, opportunities, and risks of VR application), and suggestions for implementation considerations, comprising 18 inductively derived subcategories.

M.G. and L.H. performed the content analysis of the verbatim transcribed (noScribe AI-powered Audio Transcription Version 0.6, https://noscribe.de) audio recording (Limenamics (Smart Digital Voice Recorder ZD68-32G, Limenamics, n.d.) of the workshop-based focus group supported by computer-assisted qualitative data analysis software (MAXQDA 24, VERBI–Software. Consult. Sozialforschung. GmbH, Berlin, Germany) and MS Excel (Microsoft Cooperation 2015). After familiarisation with the material, the two researchers deductively categorised the participants’ statements according to the SWOT scheme, plus the additional category ‘suggestions for implementation’. Three to four subcategories of each theme were inductively developed from the participants’ contributions (Figure 1). In an iterative process, the researchers analysed the transcript line by line, paraphrased the verbal and written contributions, and assigned them to the preformed categories. Within each SWOT category, inductive subcategories were developed through constant comparison until no additional characteristics were identified. We agreed on the name and definition of the subcategory, and provided an example quote for each subcategory for clarification for the recoding. Inconsistencies between the two coders were reconsidered and alternative interpretations incorporated into the analysis. To contextualize the content analysis, we also examined patterns of interaction, agreement, disagreement, and participation across stakeholder groups. Interim results of the analytic process were discussed with the interdisciplinary working group at weekly meetings with special attention to divergent topics.

## 3. Results

Of the 20 individuals approached via leaflets, 17 via email, and 3 by telephone, 10 confirmed their participation. A total of eight participants attended the focus group, with three joining online and five on-site (Table 2). Two patient stakeholders and one relative preferred to participate online due to health risks (1) and personal preferences (2). Reasons for declining participation included communication difficulties (nurses) and lack of interest (self-help groups) in the VR study. Two physicians who had initially confirmed their attendance did not attend, as they had forgotten the scheduled session. All participants were experienced in dealing with seriously ill patients, and they had already gained experience with VR and its application in private or clinical contexts. Participants came from a diverse range of professional backgrounds, providing a breadth of perspectives. The total duration of the focus group was 95 min.

Contributions and interactions between moderators and participants were analysed. In 40% of the cases, the participants initiated the conversation, and the moderators answered questions or provided further information; in the remaining cases (60%), the moderators initiated the conversation by asking open questions either to all or to a specific participant. Within the group of participants, interactions occurred between all participants. Most contributions were made by CAR1 and PHYS, whereas the longest contributions were made by LEU2. The fewest and shortest contributions originated from CAR2, PAB2, and LEU1 who, however, paid attention, asked questions (CAR2), and provided opinions (CAR2, PAB2), ideas, and suggestions for implementation (PAB2, LEU1) encouraged by a moderator.

The analysis resulted in 5 main themes and 18 subthemes based on the SWOT model (Figure 1).

### 3.1. Strengths of Individualised VR

This section highlights the key strengths identified from the focus group analysis on individualised VR for palliative patients, reflecting aspects that were positively received and supportive of patient well-being.

The participants highlighted the patients’ distraction from the hospital atmosphere and personal illness status. They observed positive emotions and improved general well-being during and after VR application. Further, they appreciated the versatility of VR in the clinical setting.

*“So, on my ward, based on what the patients definitely told me afterwards, the experience was entirely positive. Everyone was happy to have seen these images.”*

(physician)

Two other participants considered generic VR videos a helpful support for both patients (distraction, relaxation) and nurses (facilitating treatment in relaxed patients) during long and unpleasant bandage changes after visceral surgeries or bone marrow punctures.

*“That wasn’t so bad as support at all. […] And also, what was well received was that the films are a bit longer, it’s now less about the individualised at-home aspect, but actually more about this distraction aspect, seeing something different, getting out a bit, exactly. So, overall, the experiences weren’t bad.”*

(nurse 3)

### 3.2. Weaknesses of Individualised VR

Weaknesses focused on practical and logistical challenges that may limit the effective implementation of individualised VR for palliative care.

In this context, the limited availability of VR headsets and a limited selection of VR content was perceived as a weakness by participants in the self-help group. Nurses also reported that patients quickly became bored with standardised VR videos, which reduced their potential for effective distraction.

*“The problem was simply that the patients are with us for a very long time, that means, the first time it was okay, the second time there was nothing left to offer. That was very unfortunate, because a bit of variety would of course have been nice.”*

(nurse 3)

Further disadvantages of VR mentioned were of a more technical nature. For instance, nurses reported that some patients found the VR HMD too heavy and uncomfortable, especially when combined with normal glasses. In addition, patients who were more familiar with VR were reported to criticize the low resolution of some generic VR material. Another challenge was the visible part of the 360° video depending on the patients’ body position.

*“But the fact is really that what you see depends on your position. That means, if I see a beautiful landscape here and then lie down, I see the blue sky above, which is very nice, but it doesn’t really distract me.”*

(nurse 1)

The physician and one nurse identified the considerable organisational and time demands associated with producing and delivering individualised VR content as a key weakness. Practical challenges arose when relatives were unable to visit the hospital to collect and return the action camera (GoPro MAX, GoPro, Inc., San Mateo, CA, USA) used to record 360° videos of patients’ homes. Participants also noted that, in the context of chronic staff shortages, nurses and physicians may have limited time available to edit, prepare, and deliver personalised VR experiences. These logistical demands are particularly relevant in palliative care, where patients may have limited life expectancy and often require physical assistance to use the VR headset.

*“What I also experienced as a weakness was the great effort involved. So, I was involved in it, and also the relatives, and on my ward, there are definitely often patients who come from far away or whose relatives come from far away. And if it’s supposed to be individualised, that was often a problem. So first, someone comes to visit who could make the video, then the camera had to be there, which was also logistically difficult, and then it also took quite a long time until it was ready.”*

(physician)

### 3.3. Opportunities for Individualised VR

This section outlines the opportunities discussed in the focus group, showing areas where individualised VR interventions could be further developed or applied.

Most participants in the focus group showed a positive and open-minded attitude towards the VR application in severely ill patients.

*“I also think that this [VR] can really help.”*

(nurse 3)

All nurses were convinced that the VR application is a powerful tool that can help to emotionally support seriously ill patients. They expressed that they would like to establish routine VR use on their wards and expand the VR offer.

*“So, I can imagine definitely that the patients would benefit from it and accept it. Especially those who are there for a long time, sometimes weeks or even months.”*

(nurse 2)

Furthermore, one of the participants from leukaemia aid emphasised the relaxing and energising effect of VR on patients; the participant concurred that the individualised VR could awaken positive memories and memory-linked positive emotions. In addition, the virtual contact with relatives and friends seemed to strengthen and stabilise the patients emotionally.

*“It could also lead to relaxing. […] So, I do believe it, I can understand that this is a great approach, a great situation, that you can positively influence things with such methods.”*

(patient, leukemia aid 2)

### 3.4. Risks of Individualised VR

The physician in the focus group reported that some nursing staff on her ward held a generally negative view of the VR intervention. Their concerns centred on the possibility that VR might adversely affect patients’ well-being or condition through intensifying feelings of homesickness, longing, and sadness, which overshadowed consideration of the potential benefits and opportunities.

*“For example, on my ward, the nursing staff is very, very critical of the whole thing […] They say it worsens the longing […] There is actually no view of the possibilities it offers.”*

(physician)

*“Perhaps the longing is intensified, and one might become especially sad when seeing home and not being able to be there.”*

(nurse 2)

Concerns about potential stress and home-related content being emotionally burdensome were also discussed, although none of the focus group participants actually observed these effects.

*“I rather see the risk that this stress level simply increases. […] I also see the situation with things that come from the home environment as something that could potentially be a burden.”*

(patient, leukemia aid 2)

In contrast, motion sickness was perceived as a more manageable risk. The physician reported only a few patients experienced mild, transient dizziness and nausea; in one case, a patient declined to participate in the VR study because of previous motion-sickness-related problems. Participants noted that concerns about adverse effects may lead some patients to opt out of VR interventions, particularly those who are prone to motion sickness or depression.

*“With motion sickness, I also had a patient who said from the start that he had already tried a VR headset and it makes him feel sick.”*

(physician)

Participants expressed differing views regarding the extent to which patients and their relatives should be involved in decisions about the use of individualised VR. Most healthcare professionals and lay participants argued that the decision should primarily rest with the patient, while relatives should provide consent only where necessary, for example when recordings were made in their homes. In contrast, one participant from a patient aid organisation suggested that relatives should play a greater role in decision making to protect the patient from potential emotional distress:

*“One should really also talk to the relatives, who know the person, to ask whether this would be more of a burden or trust their judgement.”*

(patient, leukaemia aid 2)

Concerns were also raised regarding the security and privacy of personalised VR video material. One nurse expressed concern that sensitive personal information would be protected, identifying data security as an important consideration for further implementation, particularly in light of European data protection regulations:

*“Someone makes a video of my home and gives it somewhere, and I’m not entirely sure what could theoretically happen with it? Who gets this video in their hands?”*

(nurse 1)

### 3.5. Suggestions for Implementation of Individualised VR

This section summarizes the suggestions for implementation proposed by focus group participants, highlighting practical recommendations and ideas to optimize the use of individualised VR for palliative patients.

One nurse suggested streamlining the logistics of video transfer by asking relatives to upload personalised videos to a secure online platform, allowing hospital staff to access and download the files for editing. This highlighted differing views among participants regarding the balance between practical accessibility and data protection:

*“So, I can only think of it if you want to make it faster, that you explain it to the people and also say that the video can be uploaded to a Dropbox or something. So that the transfer would be easier […]”*

(nurse 1)

Regarding future technical developments, participants from patient self-help groups suggested using VR for 3D video calls. They believed that interacting with friends and relatives in a three-dimensional virtual environment could evoke stronger positive emotions and enhance the immersive nature of the experience.

Participants identified several strategies to support awareness and implementation of the VR intervention. These included organizing information events for healthcare staff that combined evidence, practical demonstrations, and discussion of the potential benefits and risks of VR use. Participants also recommended engaging specialist oncology nurses, patient support groups, and hospice services to raise awareness among patients and relatives, as well as promoting the intervention through the hospital website:

*“Why don’t you organize an event for the colleagues on the ward, show the results, also show a bit from the literature, what other people have already done, it doesn’t necessarily have to be related to oncology patients, but what opportunities are in it.”*

(nurse 1)

## 4. Discussion

This workshop-based focus group explored patient, public, and healthcare professional perspectives on the implementation of individualised VR in palliative cancer care. Using a SWOT-informed framework analysis, we identified key implementation considerations relating to emotional benefit and risk, staff engagement, logistics, data protection, and resource requirements. The findings provide practical insights into how individualised VR interventions may be implemented in palliative care settings and highlight factors likely to influence their acceptability and feasibility.

Since the conceptualisation of the study, numerous studies on VR in palliative care have been published. Apart from our project, however, only Nwosu and colleges [21] explicitly included PPI to inform the implementation strategy. They used a modified World Café approach and identified ten key questions, which closely align with the subthemes generated by our focus group.

### 4.1. Strengths

As a key strength of individualised VR interventions, focus group participants reported that patients experienced positive emotions and improved well-being. This is consistent with our own findings from the VR intervention [26] and findings from recent meta-analyses, which showed that VR can reduce psychological symptoms such as anxiety and depression, and improve well-being and quality of life [10,11,17].

One participant highlighted the role of distraction in VR interventions. Distraction is described in the literature as a central mechanism of VR, allowing patients to focus on pleasant stimuli rather than distressing symptoms [14,16,19,26,40].

### 4.2. Weaknesses

Focus group participants reported technical and logistical challenges, particularly regarding the production and recording of individualised VR content. They noted that creating individualised VR videos was resource intensive and time consuming. This observation also aligns with issues raised by Nwosu and colleges [21], who also included PPI in their study. They highlighted questions regarding responsibility for delivery and maintenance of VR, such as who ensures appropriate use of VR, who conducts assessments, and whether clinical staff are planned to be involved in its use. One of their unanswered questions was about “supporting evidence”, which pertains to the need for literature on VR’s effects and potential risks. It is important for informing both prospective participants and clinical staff, supporting education about the possibilities of VR, as well as addressing potential misconceptions or biases. Providing information and education for healthcare staff may be particularly important given reservations about VR reported by the physician participant. Participants also highlighted the need to demonstrate that the potential benefits of individualised VR justify the additional workload associated with producing, editing, and delivering personalised content within already resource-constrained care settings. From an implementation perspective, successful adoption is likely to depend not only on staff recognizing the value of the intervention, but also on whether the additional work required can be integrated into routine practice. This may help explain why some staff expressed greater hesitation towards individualised VR than has been reported for standardised VR interventions [21].

### 4.3. Opportunities

Participants saw the potential for VR to provide emotional support by giving patients the opportunity to connect with family and access the feeling of being in familiar environments. Individualised VR was considered valuable for maintaining social connections and enhancing a sense of presence despite physical separation, thereby easing isolation and improving mood. These observations are supported by Mukai and colleges [41], who also investigated the use of individualised VR videos of patients’ own homes and family. Their analysis identified key themes: relief from the hardships of hospitalisation through feelings of safety and closeness with family, reconnecting with everyday life, and the sensation of being in the same space as family members. These findings suggest that individualised VR can address the desire to be at home to a certain degree, making patients feel safe and comfortable, thus strengthening its potential to enhance emotional well-being in palliative care.

### 4.4. Risks

Although growing evidence shows that VR interventions in palliative care are generally safe with respect to psychosocial and spiritual harm [42] and can effectively reduce depressive mood and anxiety [10,11,17,43,44], little is known about the effects of individualised VR approaches. Throughout the course of the project, concerns about homesickness and other symptoms of distress were repeatedly mentioned by representatives of the patient advisory board and reported in the initial interviews of the first phase with relatives and patients [25]. In addition, the physician participating in the focus group reported that staff expressed concerns that the intervention might intensify feelings of longing. Therefore, homesickness, as well as feelings of sadness and anxiety following the perceived separation and isolation after the intervention, should be considered a potential risk. In our intervention study, we also found indications of homesickness-like responses in some individuals exposed to individualised VR content, although in most cases no signs of emotional distress or feelings of sadness or anxiety were observed [26]. Mixed results were also observed by Mukai and colleges [41], who also tried an individualised approach and found not only positive effects but also a key theme called ‘loneliness’ arising from the awareness of being physically separated from loved ones. They observed that patients were thought to experience distress when confronted with the reality of their own home environment. Thus, viewing VR content of familiar people and environments not only provides comfort and a sense of security but potentially emotional distress. Overall, this preliminary information suggests that individualised VR videos may support the well-being of palliative care patients, while the perceived gap between the virtual experience of home and the reality of the hospital setting and end of life may lead to unintended negative consequences and harms. Calman hypothesised the perceived quality of life to be a gap between a person’s desired situation and her or his actual experience. The individualised VR may—for some patients or in some situations—reduce the perceived gap between desired and actual life experience temporarily, but for others it may increase awareness of the gap [45].

Thus, the immersive nature of individualised VR may contribute to both its benefits and risks. On the one hand, virtual exposure to familiar people, places, and environments may provide comfort, familiarity, and temporary relief from the burden and isolation of hospitalisation. On the other hand, the return to the clinical setting after the VR experience may accentuate feelings of separation, loss, or sadness for patients. For individuals receiving palliative care, home may represent not only a physical place but also a symbol of autonomy, identity, and a life less constrained by serious illness. By creating a vivid sense of presence, individualised VR may strengthen these positive associations while simultaneously highlighting the contrast between the virtual experience and the patient’s current circumstances. The same features that make individualised VR emotionally meaningful may therefore also create the potential for emotional distress.

Although some participants of the focus group expressed concerns about motion sickness and negative attitudes toward VR, these appear to be uncommon. The literature indicates good tolerability, high participation rates, and strong acceptance of VR interventions among palliative care patients [23,46,47]. Multiple studies have demonstrated that VR is feasible in palliative care, with low dropout rates and minimal side effects [10,12,16,21,48]. Focus group participants also raised concerns regarding data protection. The individualised VR recordings may include highly sensitive material from patients’ homes, pets, or family members, requiring careful handling to ensure privacy and security. As highlighted in a recent scoping review [49], robust data protection is essential. The authors suggest compliance with internationally recognised standards, integration of advanced solutions, and cybersecurity training within healthcare institutions to minimise risks and maintain trust. Regarding the various concerns related to potential negative emotional consequences, such as feelings of longing, homesickness, or emotional distress, as well as concerns about data privacy and physical side effects such as motion sickness, it appears likely that self-selection may occur, as highlighted by one of the participants during the focus group discussion.

### 4.5. Suggestions for Implementation

Participants emphasised that decisions regarding informed consent for individualised VR content should be made by patients rather than their relatives. However, relatives’ concerns should be considered, as they may indicate relevant exceptions to the beneficial effects of VR in individual cases or reflect relatives’ own concerns that require consideration within palliative care.

Participants also suggested ideas such as VR-based video calls, which could represent an innovative and promising approach.

### 4.6. Limitations

The findings should be interpreted in light of several limitations. First, the study was conducted at a single centre and involved a small, heterogenous sample, which limits the transferability of the findings. Despite repeated recruitment efforts, participation remained modest, and some stakeholder groups were represented by one individual. In addition, neither relatives nor patients who had directly participated in the VR intervention were included in the focus group.

Second, some participants drew on previous experiences with VR outside the study when discussing benefits, risks, and implementation considerations. Consequently, some views may reflect broader perceptions of VR rather than experiences specific to the individualised intervention evaluated in this project.

Third, some staff interviewed had prior involvement with PPI through research or education, and several moderators were already involved in the VR project and known to participants. While this may have introduced the potential for confirmation bias, divergent views and concerns were openly discussed during the workshop.

Despite these limitations, the study provides valuable stakeholder perspectives on the implementation of individualised VR interventions in palliative care and identifies practical considerations that may inform future research and service development.

### 4.7. Implication for Future Practice/Research

For successful implementation, patients should be actively involved in the design of VR interventions, as their input helps ensure that it meets their needs and increases acceptability and effectiveness in practice.

Regarding clinical practice, the findings indicate that individualised VR cannot be delivered without additional staff resources in palliative care. The intervention requires informing patients, involving relatives, recording and editing videos, and conducting sessions with appropriate patient positioning, headset application, as well as cleaning and maintenance. Concerns expressed by patients, staff, and relatives must be addressed through clear communication, as they may otherwise hinder acceptance and implementation. The results further highlight the need to optimise and, where possible, automate processes.

From a research perspective, larger studies are needed to examine whether the added value of individualised VR content justifies the potential risks and required resources. Future work should evaluate the effectiveness of individualised VR compared to standard VR approaches and further investigate the tension between beneficial effects and experiences of separation, homesickness, or emotional distress, particularly in this vulnerable patient group. In addition, implementation research is required to better understand how VR interventions can be integrated into routine palliative care settings. Our findings raise practical considerations for organisations considering VR use in palliative care. While clinical use appears feasible and safe, further evidence on benefits, effectiveness, and practicality is needed for evidence-based recommendations. From an implementation science perspective, frameworks such as normalisation process theory would be helpful to explore how healthcare professionals make sense of individualised VR, how responsibility for its delivery is negotiated, and what organisational conditions are required for it to become embedded into routine palliative care practice [50]. This theoretically supported approach could also help examine perceived benefits and value for staff, willingness to invest time and effort, potential effects on clinical workflows, training requirements, and the overall perceived advantage of individualised VR.

## 5. Conclusions

Involving patient stakeholders and healthcare professionals in planning the implementation strategy highlighted several issues requiring attention. Clear information should be provided not only to patients but also to relatives and hospital staff to address misconceptions and improve understanding of available options. In addition, robust data protection, sufficient staffing, and more practical processes are essential for the successful implementation of individualised VR interventions.

## Figures and Tables

**Table 1:** Schedule of the workshop-based focus group.

No.	min	Programme	Performed by
1.	10′	Welcome and introduction of all participants and moderators	All
2.	5′	Written informed consent	Participants
3.	3′	Rationale and aims of the VR study	MOD 1 + 2
4.	15′	1st SWOT brainstorming on VR for hospitalised palliative patients - Collection and visualisation of the contributions on flip charts	Participants MOD 1,3,4
5.	7′	Presentation of the study results: prior patients’ and relatives’ wishes and concerns regarding VR in hospital [25] and their actual experiences with the intervention [26] - Well-being (MDBF) [35,36] - Symptoms (IPOS) [37] - VR-related symptoms (SSQ; SPES) [38,39] - Subjective VR benefit [26] - Patients’ statements about the VR experience supplementing the quantitative study results [26]	MOD 2
6.	40′	2nd SWOT brainstorming on VR for hospitalised palliative patients with a focus on questions, ideas, and suggestions for implementation - Collection and visualisation of the contributions on flip charts	Participants MOD 1,3,4
7.	3′	Acknowledgement and farewell	MOD 1

SWOT = strengths, weaknesses, opportunities, and threats; VR = virtual reality; IPOS = Integrated Palliative Care Outcome Scale; MDBF = Multi-Dimensional Well-Being Questionnaire (Mehrdimensionaler Befindlichkeitsfragebogen); SSQ = Simulator Sickness Questionnaire; SPES = Spatial Presence Experience Scale.

**Table 2:** Participant roles of the focus group. Two patient self-help participants and a patient advisory board member participated online. MOD1 was already known to most participants.

Speaker	Occupation	Role
PHYS	Physician (Palliative Care)	Participant
CAR1	Nurse (Visceral Surgery)	Participant
CAR2	Nurse (Radiology)	Participant
CAR3	Nurse (Haematology)	Participant
PAB1	Patient Advisory Board	Participant (online)
PAB2	Patient Advisory Board	Participant
LEU1	Leukaemia Aid	Participant (online)
LEU2	Leukaemia Aid	Participant (online)
MOD1	Physician (Palliative Care)	Moderator: mediation, keeping the conversation going
MOD2	Scientific assistant	Moderator: presentation of results, audio recording
MOD3	Doctoral student	Moderator: SWOT assistance
MOD4	Scientific coordinator	Moderator: Field notes

## Data Availability

The datasets generated during the current study are not publicly available due to sensitivity but are available from the corresponding author on reasonable request.
